# Exploring Glaucoma: From Pathogenesis to Emerging Diagnostic and Management Strategies

**DOI:** 10.1155/joph/8476785

**Published:** 2025-07-31

**Authors:** Rajesh Kumar Goit

**Affiliations:** Department of Ophthalmology, UCLA Jules Stein Eye Institute, Los Angeles, California 90095, USA

## Abstract

Glaucoma is a leading cause of irreversible blindness globally, affecting millions of individuals. It encompasses a group of progressive optic neuropathies characterized by retinal ganglion cell loss and visual field deterioration, often associated with elevated intraocular pressure. Despite advances in understanding the disease, glaucoma management remains challenging due to its complex pathophysiology, heterogeneous presentations, and the need for lifelong treatment. Given the rapidly evolving nature of glaucoma research and its multidisciplinary scope, there is a compelling need for a comprehensive review that synthesizes the latest findings, highlights key advancements, and identifies areas requiring further investigation. This review aims to serve as a comprehensive resource for ophthalmologists, researchers, and healthcare providers by offering an overview of glaucoma classification, pathophysiology, risk factors, diagnostic tools, and management options.

## 1. Introduction

Glaucoma is one of the leading causes of permanent blindness globally, affecting approximately 80 million people [1] and posing a significant public health challenge. It is projected that by 2040, the global prevalence of glaucoma will reach 111.8 million, up from 76 million in 2020 [2]. It encompasses a group of conditions that, despite diverse presentations and underlying mechanisms, ultimately result in damage to retinal ganglion cells (RGCs), optic nerve (ON), and progressive vision loss. The complexity of glaucoma's pathophysiology, coupled with the diverse types of glaucoma, has led to a broad and continuously expanding body of research focused on understanding its causes, risk factors, diagnostic strategies, and therapeutic options [3].

Glaucoma has a long history that dates back to ancient times. The term “glaucoma” comes from the Greek word “glaukos,” meaning “blue-green” or “gray,” which refers to the characteristic appearance of the eye in advanced stages of the disease, often with a cloudy or bluish tint. Early references to eye diseases resembling glaucoma can be found in Egyptian, Greek, and Roman medical texts [4], although the condition was not well understood in these periods. In the 18th century, advances in ocular anatomy and the development of more precise diagnostic techniques, such as the invention of the ophthalmoscope in 1851, allowed for better recognition and study of the disease. The link between elevated intraocular pressure (IOP) and glaucoma was identified in the 19th century, and over the 20th century, treatments evolved from basic surgical methods to the development of pharmaceutical agents that help lower IOP.

While substantial advancements have been made in recent years, significant gaps remain in translating this knowledge into improved patient outcomes [5]. These challenges include difficulties in early diagnosis, limitations of current treatment options, and the need for more effective neuroprotective strategies. Although IOP is a key modifiable risk factor, advancements in imaging, biomarkers, and genetic studies are transforming our ability to diagnose and manage various types of glaucoma effectively. Continued research into the underlying mechanisms and risk factors of each glaucoma subtype is crucial for developing personalized treatment approaches and improving patient outcomes. Furthermore, novel diagnostic technologies, genetic insights, and emerging therapies, such as minimally invasive glaucoma surgeries (MIGS) and artificial intelligence (AI) applications, hold great promise. However, they require critical synthesis and evaluation to fully understand their impact on clinical practice.

This review examines glaucoma, including its classification, risk factors, pathophysiology, neuroprotective mechanisms, genetics, diagnosis, and treatment, as well as ongoing challenges and future directions in management.

## 2. Overview of Glaucoma Types and Classification

Glaucoma is a group of progressive optic neuropathies characterized by damage to the ON and loss of RGCs, leading to irreversible vision loss. It is primarily classified based on anatomical and pathophysiological characteristics, particularly the drainage angle (where the cornea and iris meet) and the presence or absence of elevated IOP. In open-angle glaucoma (OAG), the drainage angle remains open, but the trabecular meshwork (TM), the eye's drainage system, becomes less efficient over time, causing a gradual increase in IOP. OAG typically develops slowly and may not cause noticeable symptoms until significant damage to the ON has occurred. Approximately 70%–90% of all glaucoma cases are OAG. Angle-closure glaucoma (ACG) occurs when the drainage angle becomes blocked or narrowed, often causing a sudden increase in IOP and rapid, severe symptoms. Both OAG and ACG can occur as primary conditions. Secondary glaucoma may develop due to factors such as trauma, medications (e.g., corticosteroids), inflammation, tumors, or conditions like pigment dispersion or pseudoexfoliation.

Pediatric glaucomas, including primary congenital glaucoma, are distinct in etiology and presentation. This form results from developmental anomalies of the anterior chamber angle (ACA), typically arising from neural crest–derived tissue dysgenesis, and leads to elevated IOP in infancy, manifesting as buphthalmos, corneal edema, and ON damage [6]. Secondary developmental glaucomas are often associated with ocular or systemic syndromes (e.g., Axenfeld–Rieger syndrome, Peter's anomaly) that further disrupt aqueous outflow [7].

The classification of glaucoma helps in understanding its diverse etiologies and guides targeted diagnostic and therapeutic strategies (Algorithm 1).

## 3. Risk Factors and Epidemiology

Demographic risk factors for glaucoma include age, gender, race, and family history, all of which influence the likelihood of developing or progressing the disease. These factors help clinicians identify patients who may require more frequent monitoring or earlier interventions. Glaucoma incidence increases with age, especially after 40, with POAG typically affecting those over 60. However, early-onset glaucoma can also occur in younger, genetically predisposed individuals [8]. Women may have a slightly higher risk of developing glaucoma, particularly in older age, due to factors such as hormonal changes during menopause [9]. A family history of glaucoma, particularly in first-degree relatives, is a major risk factor, especially for POAG [10].

The prevalence of PACG is highest in Asian populations [2]. In the United States, glaucoma is more prevalent among African Americans, Latinos, and Asian Americans [11]. African Americans, in particular, are at a significantly higher risk for POAG compared to European Americans [12].

Individuals from lower socioeconomic backgrounds often face limited access to healthcare, which can lead to delayed diagnoses and more advanced stages at detection [1]. Lower socioeconomic status is also linked to poor treatment adherence and limited access to diagnostic tools. Additionally, individuals with lower education levels may have poorer glaucoma awareness and reduced eye care utilization. Those living in rural or underserved areas face similar challenges due to limited healthcare resources, increasing the risk of undiagnosed or poorly managed glaucoma.

People with hyperopia (long-sightedness), especially those of Asian descent, are at higher risk for ACG due to the anatomical structure of their eyes. Individuals with hyperopia often have shallower anterior chambers and smaller corneas, which can make them more prone to ACG. Hypertension and poor cardiovascular health can reduce ocular blood flow, increasing the risk of glaucoma and OND [13]. Diabetes, particularly with poor blood sugar control, raises the risk due to a higher likelihood of eye conditions like diabetic retinopathy and nerve damage [14]. Severe myopia increases the risk of POAG due to anatomical changes in the eye [15]. Taxane-based chemotherapy drugs (e.g., paclitaxel and docetaxel) may increase IOP, potentially leading to taxane-induced glaucoma [16].

In addition to these common risk factors, several less common conditions can also contribute to glaucoma development. For instance, amyloid deposits, often associated with neurodegenerative diseases like Alzheimer's, can accumulate in the TM, disrupting aqueous outflow and contributing to glaucoma [17]. Lowe syndrome, a rare genetic disorder, is also linked to glaucoma due to its impact on ocular development [18]. Furthermore, *Onchocerca volvulus*, the parasitic worm responsible for onchocerciasis, can induce a form of secondary glaucoma, particularly in regions where the parasite is endemic [19, 20].

## 4. Genetic and Epigenetic Contributions

Observational research has demonstrated that both the onset and progression of glaucoma have a significant hereditary component. Genome-wide association studies have identified over a hundred genetic markers linked to increased glaucoma risk [21]. The disease is influenced by both polygenic inheritance, where multiple genetic variants contribute to risk, and monogenic mutations that cause inherited forms of glaucoma. Polygenic risk scores, which aggregate these genetic variants, can predict an individual's susceptibility, aiding early detection and personalized treatment, especially when integrated with clinical factors such as age and IOP [22]. Lifestyle factors, such as diet and smoking, also interact with genetic predispositions, influencing the disease's onset and progression [23]. Certain genetic variants may predispose individuals to environmental stressors like oxidative stress, which can accelerate glaucoma development. Advanced sequencing technologies, including whole-exome and whole-genome sequencing, are unveiling rare genetic variants that may have a stronger influence on disease risk compared to more common variants. These genetic alterations affect various biological processes, including retinal cell function, ON health, eye development, inflammation, and vascular regulation.

The MYOC gene is well studied for its link to both juvenile- and adult-onset POAG [24], with mutations causing misfolded myocilin that disrupt aqueous outflow. Mutations in the OPTN and TBK1 genes are associated with NTG, with both affecting autophagy and inflammation, leading to RGC death [25]. Other genes, such as CYP1B1, FOXC1, and WDR36, contribute to POAG [26], while PITX2 and FOXC1 mutations impair anterior segment development [27], increasing susceptibility to PACG. Variants in PLEKHA7 and COL11A1 also increase PACG risk by affecting the ACA and iris thickness [28]. Additionally, MMP9 is involved in extracellular matrix remodeling in the TM, contributing to ACG pathogenesis [29]. CYP1B1 and TEK mutations cause primary congenital glaucoma by disrupting the TM and Schlemm's canal, respectively [30]. Finally, OPA1 and MFN2, which regulate mitochondrial fusion and axonal transport, are implicated in glaucoma through disruption of mitochondrial dynamics, compromising the long-range energy supply critical for RGC function [31].

Epigenetics plays a significant role in the development and progression of glaucoma. Epigenetic mechanisms, such as DNA methylation, histone modification, and noncoding RNA regulation, can influence gene expression without altering the underlying genetic code. In glaucoma, these epigenetic changes can affect key pathways involved in the regulation of IOP, RGC survival, and ON degeneration. For example, abnormal DNA methylation patterns may alter the expression of genes involved in cell death and inflammation, which are critical processes in glaucomatous damage [32, 33]. Additionally, environmental factors like oxidative stress, aging, and increased IOP can modify epigenetic marks, further contributing to the pathophysiology of glaucoma. Understanding the epigenetic modifications associated with glaucoma could offer new insights into its prevention, diagnosis, and treatment, potentially leading to targeted therapies that address both genetic and epigenetic factors in the disease.

## 5. Pathophysiology of Glaucoma

### 5.1. IOP and Glaucomatous Damage

IOP is the fluid pressure inside the eye, crucial for maintaining its structure and function. It results from the balance between the production and drainage of aqueous humor (AH), the fluid that nourishes the eye's avascular structures (e.g., cornea, lens). AH is produced by the ciliary body epithelium and secreted into the posterior chamber, which is bounded posteriorly by the lens and zonules of Zinn, and anteriorly by the iris. It then flows through the pupil into the anterior chamber, bordered posteriorly by the iris and anteriorly by the cornea. It exits via two pathways. The conventional (trabecular) outflow pathway involves drainage through the TM into Schlemm's canal and then into the episcleral veins, accounting for 70%–90% of total outflow. The unconventional (uveoscleral) outflow pathway allows AH to pass through the ciliary muscle into the suprachoroidal space, where it is absorbed by scleral tissues or lymphatic vessels. This route contributes 10%–30% of the total aqueous outflow.

**Figure 1:**
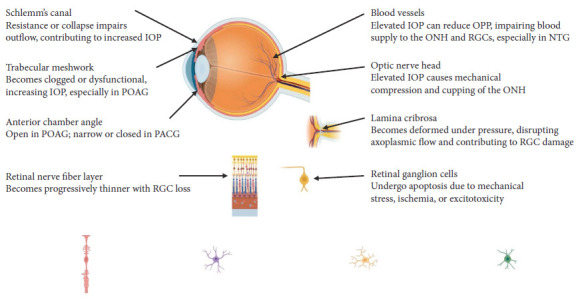
In glaucoma, multiple ocular structures—including glial cells—are affected, primarily due to elevated IOP and/or impaired ocular blood flow. IOP, intraocular pressure; NTG, normal-tension glaucoma; ONH, optic nerve head; OPP, ocular perfusion pressure; PACG, primary angle-closure glaucoma; POAG, primary open-angle glaucoma; RGC, retinal ganglion cell.

Glaucomatous damage related to IOP involves mechanical, vascular, and molecular factors. Multiple ocular structures, including glial cells, are affected in glaucoma (Figure 1). High IOP directly compresses the optic nerve head (ONH), deforming the lamina cribrosa and causing optic disc cupping [34]. The superior and inferior regions of the lamina cribrosa are particularly vulnerable due to uneven stress distribution [35]. This deformation disrupts axoplasmic flow in RGC axons, leading to cellular stress and apoptosis [36]. Over time, this results in progressive thinning of the retinal nerve fiber layer (RNFL) [37]. Ocular blood flow is normally autoregulated, but elevated IOP can compress the capillaries at the ONH, reducing ocular perfusion pressure (OPP), which is determined by the difference between mean arterial pressure and IOP, and impairing blood supply to RGCs [38]. Chronic ischemia leads to oxidative stress and mitochondrial dysfunction, accelerating RGC death [38]. In NTG, glaucomatous damage occurs despite normal IOP due to impaired autoregulation of ONH blood flow, as well as systemic factors like hypotension, sleep apnea, and vascular dysregulation [39]. Elevated IOP also stretches the peripapillary sclera [40], impairing the connection between the retina and ONH, while activating mechanosensitive ion channels (e.g., TRPV4), triggering cellular calcium changes and damage [41]. Additionally, during glaucomatous stress, pericytes around retinal capillaries constrict in a calcium-dependent manner, reducing blood flow and further compromising the supply of oxygen and nutrients to the retina [42].

### 5.2. IOP Variation

Normal IOP typically ranges between 10 and 21 mmHg, with variations influenced by circadian rhythms, posture, and other physiological factors. IOP fluctuations, even within the normal range, are linked to an increased risk of glaucoma progression. The fluctuation of IOP can be classified based on the duration of monitoring [43], and understanding these fluctuations is essential for optimizing patient care and developing targeted therapeutic interventions in various ocular conditions. Instantaneous IOP fluctuations are rapid pressure changes over short durations (seconds to minutes). These reflect the eye's response to external stimuli such as blinking, body position changes, or atmospheric pressure shifts. Diurnal–nocturnal IOP fluctuation occurs over a 24-h period, with variations between day and night. Short-term IOP fluctuations occur over hours to days. These fluctuations are important for managing conditions like acute ACG or post-operative recovery. Long-term IOP fluctuations occur over weeks, months, or even years. These variations are particularly significant in chronic conditions like POAG. In this context, provocative tests such as the closed-eyelid test have demonstrated that an IOP increase in response to eyelid closure can predict the long-term risk of developing POAG, supporting the clinical relevance of assessing IOP variability beyond static measurements [44, 45].

### 5.3. IOP and Circadian Rhythms

IOP follows a circadian rhythm, regulated by the suprachiasmatic nucleus, which receives light information from intrinsically photosensitive RGCs. IOP typically peaks during the late dark period and is higher in the supine position compared to the sitting position during the light/wake period in the healthy young adults [46]. Therefore, elevated IOP during the night may cause more damage to the ON if not managed properly in people with glaucoma. Interestingly, the nocturnal elevation of human IOP coincides with the time when systemic blood pressure is at its lowest [46]. Studies have indicated a link between low nighttime blood pressure and glaucomatous OND [47, 48]. Furthermore, OPP over 24 h has been shown to be lower in younger individuals than in older ones, and the average nocturnal OPP in the supine position is higher than the average diurnal OPP in the sitting position in both age groups [49]. These findings suggest that the balance between IOP and systemic blood pressure during the night may critically affect ONH perfusion. In particular, the simultaneous occurrence of elevated IOP and reduced blood pressure may result in significant nocturnal reductions in OPP, potentially leading to ON hypoperfusion and contributing to glaucomatous damage. Thus, low nocturnal OPP may be a modifiable vascular risk factor for glaucoma progression, especially in individuals with NTG or dysregulated systemic blood pressure.

### 5.4. Molecular Pathways Leading to RGC Death

The pathogenesis of glaucoma involves complex interactions of molecular signaling, cellular responses, and tissue-level changes that contribute to the progressive loss of RGCs.

#### 5.4.1. Apoptotic Signaling Dysregulation

Apoptotic dysregulation plays a central role in this process, with both extrinsic and intrinsic apoptotic pathways becoming dysregulated. Normally, apoptosis removes damaged cells, but in glaucoma, elevated IOP, oxidative stress, and glutamate excitotoxicity trigger excessive activation of these pathways [50]. Misfolded proteins accumulate in the endoplasmic reticulum (ER) of RGCs, triggering the unfolded protein response. Chronic ER stress then activates pro-apoptotic signaling pathways [51]. Oxidative stress and mitochondrial dysfunction disrupt the intrinsic pathway, causing mitochondrial permeability transition and the release of pro-apoptotic factors like cytochrome c. Concurrently, elevated IOP activates death receptors in the extrinsic pathway, such as Fas, TNFR1, and TRAIL receptors, which trigger a cascade that activates downstream caspases (e.g., caspase-8) [52], amplifying apoptotic signals. Dysregulation of key regulatory proteins, such as members of the Bcl-2 family (e.g., Bax, Bcl-2, Bcl-xL) and caspases, results in persistent apoptosis, even without normal triggers. The overexpression of pro-apoptotic proteins like Bax, combined with reduced expression of antiapoptotic proteins like Bcl-2, exacerbates RGC loss by driving the cell toward mitochondrial-mediated apoptosis [53].

#### 5.4.2. Autophagy and Mitochondrial Dysfunction

In addition to apoptosis, other forms of cell death are dysregulated in glaucoma. Autophagy, responsible for recycling damaged components, becomes either impaired or excessive, contributing to RGC stress and death. This is often linked to mitochondrial dysfunction, oxidative stress, and neuroinflammation [54]. In glaucomatous conditions, mitophagy (the selective degradation of damaged mitochondria) may be hindered, resulting in the accumulation of dysfunctional mitochondria that exacerbate oxidative damage and cell death [55].

#### 5.4.3. Necroptosis, Pyroptosis, and Ferroptosis

Necroptosis, a form of regulated necrosis, is activated by elevated IOP and ischemia, involving inflammatory proteins like RIPK3 and MLKL, further exacerbating RGC loss [56]. Similarly, pyroptosis, an inflammatory cell death process, is associated with glial activation and the release of pro-inflammatory cytokines (such as IL-1β and IL-18), which amplify damage [57]. Finally, there may be an imbalance in iron homeostasis, leading to excess free iron that can catalyze the production of reactive oxygen species (ROS), contributing to oxidative damage and triggering ferroptosis [57].

#### 5.4.4. Glutamate Excitotoxicity

Excitotoxicity, driven by dysregulated glutamate signaling, is another crucial mechanism contributing to RGC death. In glaucoma, excessive activation of glutamate receptors, especially NMDA and AMPA receptors, results in an influx of calcium ions into RGCs. Elevated intracellular calcium levels disrupt mitochondrial function, activate pro-apoptotic signaling pathways, and trigger other forms of cell death, including necroptosis and autophagy [56]. Additionally, excitotoxicity promotes the activation of calcium-dependent enzymes (e.g., calpains) that further damage cellular structures, leading to irreversible RGC loss [58].

#### 5.4.5. Ischemia and Energy Failure

Chronic ischemia has long been implicated in glaucomatous optic neuropathy, contributing to oxidative stress, mitochondrial dysfunction, and ultimately RGCs death. However, emerging evidence suggests that mitochondrial dysfunction may play a primary, causative role in glaucoma pathogenesis [59], rather than being merely a downstream effect of ischemic injury. Age-related declines in mitochondrial function—such as impaired oxidative phosphorylation and increased production of ROS—can reduce the metabolic resilience of RGCs [60], making them more susceptible to stress even in the absence of overt ischemia. These mitochondrial changes may precede or occur independently of vascular compromise, implicating them in disease initiation.

#### 5.4.6. RAAS Overactivation and Neurovascular Stress

Excessive activation of the renin-angiotensin-aldosterone system (RAAS) can increase IOP by stimulating AH production and impairing outflow through the TM via angiotensin II type 1 receptor signaling [61]. Additionally, RAAS overactivity contributes to ONH ischemia by promoting vasoconstriction and endothelial dysfunction, particularly relevant in NTG [62]. Angiotensin II also induces oxidative stress, glial activation, and inflammatory cytokine release, exacerbating RGC apoptosis and fibrotic remodeling [61]. These mechanisms suggest that local and systemic RAAS dysregulation may contribute to both pressure-dependent and pressure-independent damage in glaucoma, highlighting the potential of RAAS inhibitors as therapeutic agents with both IOP-lowering and neuroprotective effects.

#### 5.4.7. Glial Activation

Glial cells, including Müller cells, astrocytes, microglia, and oligodendrocytes, play key roles in glaucoma pathophysiology by interacting with RGCs [63]. Müller cells become reactive under stress, contributing to inflammation and oxidative damage. Astrocytes, at the ONH, release pro-inflammatory cytokines (such as TNF-α and IL-6) and growth factors (e.g., TGF-β) in response to elevated IOP, further promoting RGC death [64]. GFAP immunohistochemistry typically highlights reactive Müller cells in both retinal layers and astrocytes in the nerve fiber layer in glaucoma patients [65]. Microglia are the resident immune cells of the retina. In glaucoma, they become activated, contributing to neuroinflammation and excitotoxicity. Activated microglia release a variety of inflammatory cytokines, ROS, and glutamate, further damaging RGCs [66, 67]. This exacerbates the cycle of inflammation and cell death. Oligodendrocytes are responsible for myelinating RGC axons in the ON. In glaucoma, ischemic damage caused by elevated IOP can impair the function of oligodendrocytes, leading to demyelination and further injury to RGC axons, which compromises axonal conduction and contributes to ON degeneration.

#### 5.4.8. Immune Dysregulation

Beyond glial-mediated neuroinflammation, glaucoma involves broader dysregulation of innate and adaptive immune pathways that contribute to RGC death [68]. A key component of this immune response is the complement system, which becomes aberrantly activated in glaucomatous tissues. Activation of complement components such as C3a and C5a can trigger an inflammatory cascade, including the recruitment of immune cells, and promote oxidative stress and neuroinflammation. Chronic activation of the complement system in glaucoma may contribute to persistent inflammation, which amplifies tissue damage, disrupts homeostasis, and accelerates the loss of RGCs [69].

In addition to innate responses, autoimmune mechanisms are increasingly recognized in glaucoma pathogenesis. Several studies have identified circulating autoantibodies against retinal and ON proteins—such as heat shock proteins [70], myelin basic protein [71], and γ-enolase [72]—suggesting a loss of self-tolerance and persistent low-grade autoimmunity. These antibodies may interfere with neuroprotective pathways or directly contribute to RGC apoptosis, particularly in forms of glaucoma not primarily driven by elevated IOP, such as NTG.

## 6. Glaucoma Diagnosis and Monitoring

Glaucoma often progresses silently, without noticeable symptoms, until significant neural damage has occurred. Early diagnosis and continuous monitoring are essential to prevent vision loss in glaucoma. Several diagnostic tests are used to assess the ON, retina, and visual pathways in order to detect glaucoma, track progression, and evaluate treatment efficacy (Algorithm 2). A combination of diagnostic tests is essential for glaucoma diagnosis and management. Structural tests (e.g., optical coherence tomography [OCT], optic disc photography) assess changes in the retina and ON, while functional tests (e.g., visual field testing, electrophysiological evaluations) evaluate the impact on vision. Regular monitoring of these parameters helps track disease progression, detect early damage, and guide treatment decisions to preserve vision.

## 7. Advancements in Diagnostic Tools

Advancements in glaucoma diagnostic tools have significantly enhanced the detection, monitoring, and management of this progressive optic neuropathy. These innovations improve diagnostic accuracy, enable earlier intervention, and help prevent irreversible vision loss. High-resolution OCT, using longer wavelengths of light, offers deeper imaging of key structures such as the ONH and peripapillary retina, especially in patients with thicker retinal layers. When combined with OCTA, this technique provides noninvasive visualization of retinal and ON vasculature, which is essential for detecting ischemia and vascular insufficiency associated with glaucoma. The integration of AI into imaging technologies like OCT and fundus photography further enhances the detection of subtle glaucomatous changes, such as RNFL thinning and optic disc cupping, while reducing human error. AI can also combine imaging results with other patient data, like IOP readings and visual field results, to predict disease progression and guide personalized treatment plans. Technological advances also offer several options for glaucoma monitoring outside of the clinical setting, including home-based tonometry and perimetry [73]. The Sensimed Triggerfish contact lens sensor enables continuous 24-hour IOP pattern monitoring. Furthermore, diffusion tensor imaging (DTI) offers an exciting frontier in glaucoma research, allowing for the visualization of microstructural damage to the ON and visual pathways, potentially aiding in early detection and monitoring disease progression. While DTI holds promise, its widespread clinical use still requires further validation and refinement.

## 8. Pharmacological Treatment

Glaucoma medications primarily aim to reduce IOP by modifying the production or drainage of AH. Prostaglandin analogs (e.g., latanoprost, bimatoprost, travoprost, tafluprost) enhance AH outflow through the uveoscleral pathway [74]. By binding to prostaglandin receptors (e.g., FP receptors), they relax the ciliary muscle and improve AH outflow through the sclera. Beta-blockers (e.g., timolol, betaxolol, carteolol) reduce AH production by binding to β1 and β2 adrenergic receptors in the ciliary body, thus inhibiting its production [75]. Alpha agonists (e.g., brimonidine, apraclonidine) bind to *α*2-adrenergic receptors on the presynaptic neurons in the ciliary body and TM [76]. This reduces AH production and, to a lesser extent, increases uveoscleral outflow. Carbonic anhydrase inhibitors (e.g., dorzolamide, brinzolamide [topical]; acetazolamide [oral]) decrease AH production by inhibiting the enzyme carbonic anhydrase in the ciliary body [77]. Rho kinase inhibitors (e.g., netarsudil) relax TM cells, enhancing AH outflow through the TM-Schlemm's canal [78]. Miotics (e.g., pilocarpine), though less commonly used today, stimulate muscarinic receptors in the ciliary muscle and iris sphincter [79]. This induces pupillary constriction (miosis), which opens the TM, enhancing AH outflow through the conventional pathway. Combination medications (e.g., cosopt, dorzolamide/timolol) enhance efficacy by targeting multiple pathways, often improving patient adherence [80]. Other pharmacological options for glaucoma include corticosteroids and neuroprotective agents. Corticosteroids, while useful for treating conditions like eye inflammation, can increase IOP and may trigger or worsen glaucoma in susceptible individuals, leading to steroid-induced glaucoma [81]. Neuroprotective agents, still in the research phase, aim to protect RGCs from damage by targeting pathways involved in oxidative stress, mitochondrial dysfunction, and excitotoxicity, though they are not yet widely used in clinical practice [82]. However, like all medications, glaucoma treatments come with potential side effects and challenges related to patient adherence. These factors are critical in determining the effectiveness and long-term success of treatment (Algorithm 3).

## 9. Neuroprotective Mechanisms and Potential Therapeutic Targets in Glaucoma

Neuroprotective strategies in glaucoma aim to protect RGCs and the ON, complementing the primary goal of lowering IOP. While current treatments focus on IOP reduction, neuroprotection targets the cellular mechanisms driving RGC and ON degeneration, potentially preventing further vision loss even with controlled IOP. Animal research has been pivotal in understanding the mechanisms of glaucomatous damage and testing potential neuroprotective agents. Studies in rodent and primate models have demonstrated that various compounds, including neurotrophic factors, anti-inflammatory agents, and ion channel blockers, can reduce RGC loss and protect the ON following elevated IOP.

### 9.1. Neurotrophic Factors and Antiapoptotic Agents

Neurotrophic factors, including brain-derived neurotrophic factor (BDNF), ciliary neurotrophic factor, and glial cell line–derived neurotrophic factor (GDNF) promote RGC survival and repair [83]. GDNF-loaded biodegradable microspheres offer significant neuroprotection in experimental glaucoma [84]. Brimonidine protects RGCs from glutamate excitotoxicity and oxidative stress [85] while N-acetylcysteine reduces oxidative stress and improves RGC survival [86].

### 9.2. Anti-Inflammatory and Ion Channel Modulators

Anti-inflammatory agents like minocycline reduce retinal inflammation [87], and COX-2 inhibitors mitigate inflammation and oxidative stress [88]. NOS inhibitors target excessive nitric oxide neurotoxicity [89]. Calcium channel blockers such as nimodipine prevent excessive calcium influx and apoptosis in RGCs [90]. Szeto-Schiller peptides stabilize mitochondrial membranes and improve energy production [91].

### 9.3. Gene Therapy and Genetic Neuroprotection

Gene therapy is another promising approach for neuroprotection in glaucoma. It involves delivering genes that encode for neurotrophic factors or other protective molecules directly to the retina for neuroprotection, or to the TM and ciliary body to improve their function and reduce IOP. Early trials have shown that AAV2-BDNF gene therapy can improve RGC survival [92]. Emerging technologies like CRISPR-Cas9 also hold potential for editing genes involved in the pathogenesis of glaucoma [93].

### 9.4. Stem Cell and Cell-Based Therapies

Stem cell and cell-based therapies offer an innovative approach to replacing damaged retinal tissue and regenerating lost RGCs. Stem cells, such as induced pluripotent stem cells or those derived from the retina, have the potential to differentiate into functional RGCs. Additionally, stem cells from the ciliary body may be induced to generate new retinal neurons, promoting retinal repair. There is also the potential for using stem cells to regenerate ON fibers, which could help restore lost visual function [94].

### 9.5. Immunomodulatory Approaches to Neuroprotection

Immunomodulatory approaches have shown potential as neuroprotective therapies. Experimental studies demonstrate that TNF-α inhibitors, complement inhibitors, and microglial modulators (e.g., minocycline) can suppress inflammation and preserve RGCs [95]. Emerging approaches such as regulatory T cell therapy and tolerogenic vaccination are under investigation to restore immune balance and prevent pressure-independent ON degeneration [96].

### 9.6. Lifestyle and Nutraceutical-Based Neuroprotection

Emerging evidence suggests that lifestyle modifications may have neuroprotective effects in glaucoma. Regular physical activity has been shown to reduce the risk of developing glaucoma and may help protect against its progression [97]. Certain dietary interventions also show potential in protecting retinal cells from oxidative damage. For example, a supplement containing three food-derived antioxidants—hesperidin, crocetin, and *Tamarindus indica—*was effective in reducing markers of oxidative stress in NTG patients with elevated oxidative stress [98].

In recent years, nutraceuticals have gained attention for their potential role in the early intervention of glaucoma. For instance, melatonin has shown IOP-lowering effects via modulation of AH dynamics and exerts antioxidant and anti-inflammatory actions that support RGC survival [99]. Forskolin, a plant-derived compound, reduces IOP through activation of adenylyl cyclase and has shown emerging neuroprotective properties [100]. Epigallocatechin gallate, a green tea polyphenol, is a potent antioxidant that mitigates oxidative stress and apoptosis in experimental glaucoma models, with preliminary clinical data supporting its benefit in ocular hypertension and glaucoma [101].

### 9.7. Clinical Evidence Supporting Neuroprotection

Clinical evidence supports the potential for neuroprotection in glaucoma patients. For example, brimonidine significantly improved impaired retinal vascular autoregulation in patients with NTG [102]. Coenzyme Q10 enhanced PERG and VEP responses in OAG patients [103] with notable effects on RNFL thickness [104], suggesting neuroprotective benefits. Topical citicoline in OAG patients increased PERG amplitude and VEP responses [105]. Additionally, citicoline therapy increased RNFL and ganglion cell complex thickness and improved standard automated perimetry mean deviation values in chronic OAG patients [106].

### 9.8. Translational Challenges and Future Directions

Since the concept of neuroprotection was introduced, numerous molecules have been tested, but with limited success in translating from laboratory studies to clinical use. Despite promising preclinical results, most have failed to progress past Phase 2 trials, and only a few have reached Phase 3 [82]. For example, while memantine is approved for treating neurodegenerative diseases such as Alzheimer's and Parkinson's, long-term use in OAG patients has not prevented glaucomatous progression [107]. As a result, regulatory approval for widespread use remains pending, with many therapies still in the clinical trial phase.

**Figure 2:**
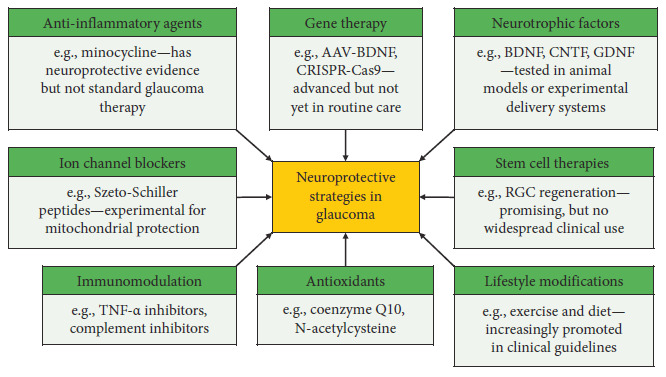
Experimental or emerging therapies in glaucoma. These are promising but mostly still in preclinical or early clinical research stages. AAV, adeno-associated virus; BDNF, brain-derived neurotrophic factor; CNTF, ciliary neurotrophic factor; CRISPR-Cas9, clustered regularly interspaced short palindromic repeats–CRISPR-associated Protein 9; GDNF, glial cell line–derived neurotrophic factor; RGC, retinal ganglion cell; TNF-α, tumor necrosis factor alpha.

Figure 2 summarizes current and emerging neuroprotective approaches aimed at preserving RGCs and ON health in glaucoma. These strategies include pharmacological agents (anti-inflammatory agents, immunomodulators, antioxidants, ion channel blockers), biological therapies (neurotrophic factors, gene therapy, stem cell therapies), and lifestyle modifications (exercise and diet). Many of these approaches remain experimental and are not yet part of routine clinical practice.

## 10. Laser Therapy in Glaucoma

Laser therapy is an important treatment option for glaucoma, particularly when medications are ineffective, poorly tolerated, or insufficient in controlling IOP [108]. In OAG, laser trabeculoplasty—including argon laser trabeculoplasty and selective laser trabeculoplasty—enhances AH outflow through the TM, offering a noninvasive alternative to surgery. For ACG, laser peripheral iridotomy is a first-line treatment that creates a small hole in the peripheral iris, relieving pupillary block and restoring ACA anatomy. Laser iridoplasty may be used adjunctively to contract the peripheral iris and widen the angle further.

In more advanced or refractory cases, cyclodestructive laser procedures such as transscleral cyclophotocoagulation and endoscopic cyclophotocoagulation are used to partially ablate the ciliary body, thereby reducing AH production. While laser therapies are effective, they may not offer long-term IOP control in all cases, and surgical options may become necessary for progressive disease.

## 11. Surgical Options for Glaucoma Treatment

Surgical interventions for glaucoma are considered when medications and laser treatments fail to adequately control IOP [109]. The most common traditional surgery, trabeculectomy, creates a drainage passage in the eye to reduce IOP but carries risks such as infection, cataract formation, and the potential need for revision surgery [110]. A less invasive alternative, nonpenetrating deep sclerectomy, offers a lower risk of complications but may not achieve as significant an IOP reduction, especially in advanced glaucoma cases [111]. For refractory or advanced cases, glaucoma drainage implants (e.g., Ahmed or Baerveldt implants) provide an alternative drainage route, while tube shunt surgery is often used after failed trabeculectomy and may require long-term management due to complications like tube obstruction [112].

MIGS are newer techniques designed to reduce IOP with fewer risks, faster recovery, and less discomfort than traditional surgeries. MIGS methods such as the iStent and Hydrus Microstent bypass the TM to improve AH outflow [113]. Other devices, like the Xen Gel Stent, create a new drainage pathway into the subconjunctival space, typically used in OAG cases that are refractory to medications or prior surgeries [114]. The CyPass Micro-Stent targets the supraciliary space to facilitate fluid drainage, while the Trabectome removes part of the TM to enhance outflow through the eye's natural system [115]. MIGS is especially suitable for mild to moderate glaucoma, offering a balance between safety and efficacy.

In certain cases, goniotomy (though more commonly used in congenital or juvenile glaucoma) can be used to address peripheral anterior synechiae or to help open the angle [116]. Cyclocryotherapy, which reduces AH production, is used for refractory glaucoma but is less common today due to the advent of more precise techniques like cyclophotocoagulation [117]. The choice of procedure depends on glaucoma severity, patient health, and the outcomes of previous treatments.

Laser therapy and surgical interventions are key treatment options when medications are insufficient to control IOP.  Table 1 summarizes the current approaches, including indications and key considerations for each procedure.

## 12. Drug Delivery Systems

Drug delivery systems in glaucoma are designed to improve the efficacy and patient compliance by ensuring sustained, localized delivery of medications directly to the eye [118, 119]. These systems address common challenges like poor drug bioavailability, frequent dosing, and ocular side effects. Examples include ocular inserts such as Ocusert, which use punctal plugs for sustained drug release, and timolol-loaded nanoparticles, which enhance drug penetration while reducing systemic side effects. Additionally, prostaglandin analog implants like the Durysta implant provide extended release of medication into the eye, significantly reducing the need for daily eye drops. The iDose TR Implant is the first FDA-approved device of its kind to deliver travoprost directly into the eye continuously, 24/7, for extended periods, marking a significant advancement in glaucoma treatment [120]. By controlling the release rate and targeting specific eye tissues, these innovative systems improve IOP reduction, minimize systemic absorption, and ultimately enhance patient adherence to treatment regimens. Advances in biomaterials and nanotechnology have further expanded the potential for more efficient, patient-friendly therapies, offering new hope for better glaucoma control [121].

## 13. Future Challenges and Research Directions in Glaucoma Management

Despite significant advances in glaucoma diagnosis and treatment, several critical challenges remain, particularly in managing advanced stages and ensuring long-term vision preservation. Current therapies, while effective in lowering IOP, often fail to prevent RGC death and ON degeneration, especially in conditions like NTG. Drug delivery faces barriers such as ocular anatomical constraints, poor patient adherence, and treatment side effects [122], while surgical interventions carry inherent risks and may not be accessible in all settings [123]. Early diagnosis remains suboptimal due to the lack of sensitive and specific tests for detecting disease at its earliest stages. Moreover, gene and stem cell therapies, though promising, face limitations related to delivery efficiency, regenerative potential, ethical concerns, and high costs, complicating their translation to routine practice [124]. Personalized treatment is further challenged by genetic heterogeneity among patients, and disparities in healthcare access continue to affect outcomes, particularly in low-resource settings. Patient education and sustained adherence to complex treatment regimens also remain vital yet difficult to achieve.

Deep learning (DL) algorithms have demonstrated strong performance in detecting glaucomatous changes from fundus photographs, OCT scans, and visual field data, even at preperimetric stages. However, real-world application presents significant challenges. External validation often yields lower accuracy than internal testing due to variability in imaging datasets, reference standards, and clinical settings. Additionally, DL models require large, high-quality annotated datasets, are susceptible to overfitting, and must undergo rigorous clinical validation before widespread adoption [125]. Addressing these unmet needs requires targeted research and global collaboration. Future investigations should prioritize genetic and epigenetic studies to uncover novel risk factors and therapeutic targets, leveraging whole-genome sequencing and diverse populations to understand gene–environment interactions and epigenetic mechanisms such as DNA methylation and noncoding RNAs. Research into the molecular pathways of RGC loss—including neuroinflammation, glial activation, mitochondrial dysfunction, and oxidative stress—will be essential for developing effective neuroprotective strategies. Innovations in early detection, such as molecular biomarkers in tear fluid or blood and advanced imaging techniques, hold promise for improving diagnostic accuracy, especially in underserved or high-risk communities. Clinical trials focusing on neuroprotective agents, improved drug delivery systems like gene or nanoparticle-based therapies, and personalized treatment approaches are critical for slowing disease progression. Expanding community-based screening, adopting AI for early diagnosis, and enhancing patient-centered care through individualized treatment plans and adherence support will further strengthen glaucoma management. Finally, continued advances in MIGS and the exploration of robotic-assisted techniques are expected to improve surgical safety and outcomes, reducing the overall treatment burden for patients worldwide.

## 14. Limitations of the Study

This review has several limitations that should be acknowledged. First, although it aims to provide a comprehensive overview of current understanding and advancements in glaucoma research and management, it may be influenced by publication bias, as studies with negative or inconclusive findings are less likely to be published or cited. Second, the selection of studies may be limited by language restrictions, database coverage, or search strategy, potentially omitting relevant research. Finally, while the review synthesizes a wide range of genetic, molecular, and clinical insights, it does not include a meta-analytic component, which would allow for a quantitative assessment of effect sizes and study heterogeneity. Future reviews incorporating systematic methodologies and meta-analyses could provide more robust, data-driven conclusions.

## 15. Conclusions

This review has provided a comprehensive overview of the current understanding of glaucoma, encompassing its pathophysiology, genetic and molecular mechanisms, diagnostic advancements, and therapeutic approaches. The multifactorial nature of glaucoma, involving IOP-dependent and -independent pathways, underscores the complexity of this neurodegenerative disease. From a personal perspective, the convergence of molecular genetics, advanced imaging, and emerging neuroprotective strategies represents a promising frontier in glaucoma research. However, despite significant progress, challenges remain in translating these findings into equitable and effective clinical care globally. Future research should prioritize longitudinal studies to validate biomarkers, explore patient-specific therapeutic approaches such as gene and stem cell therapy, and improve early detection methods. Greater interdisciplinary collaboration and public health initiatives are essential to reduce the global burden of glaucoma and to ensure that novel interventions are accessible across diverse healthcare settings.

## Figures and Tables

**Algorithm 1:**
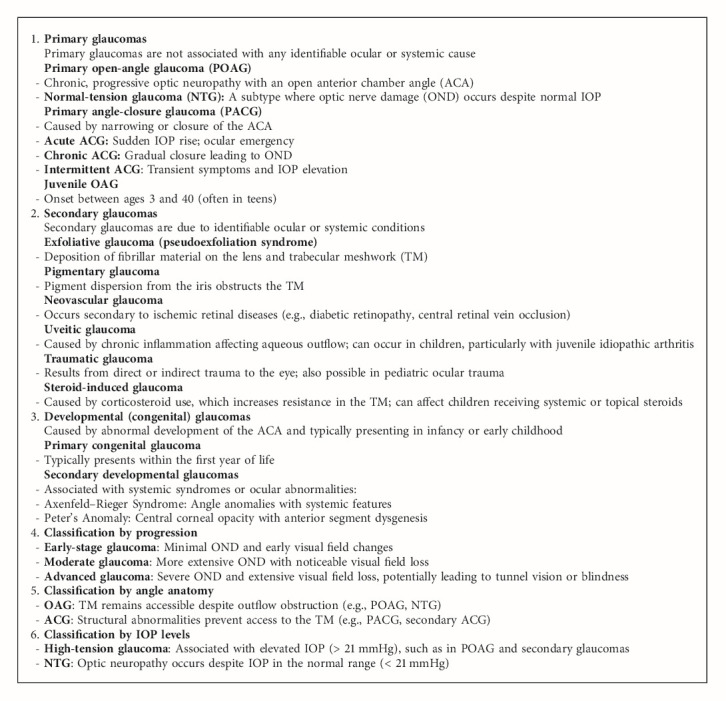
Glaucoma types and classification.

**Algorithm 2:**
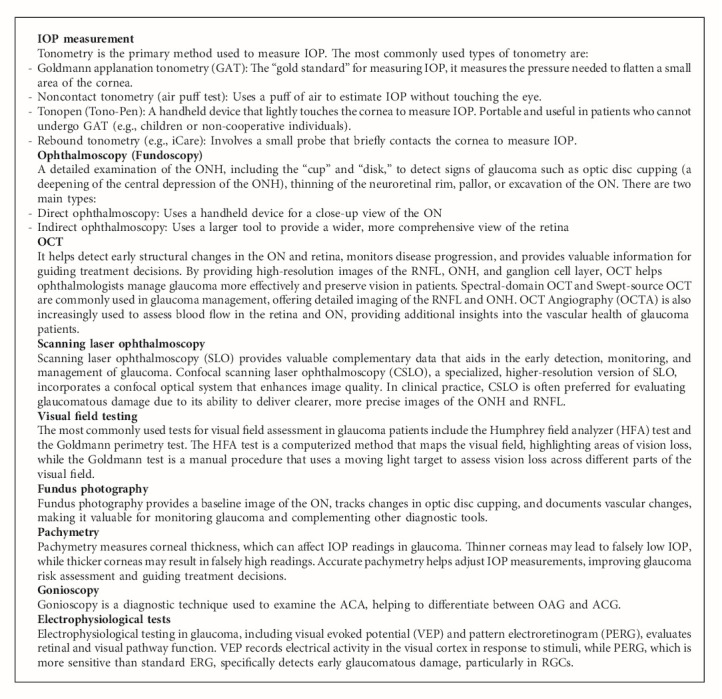
Key glaucoma diagnosis and monitoring tests.

**Algorithm 3:**
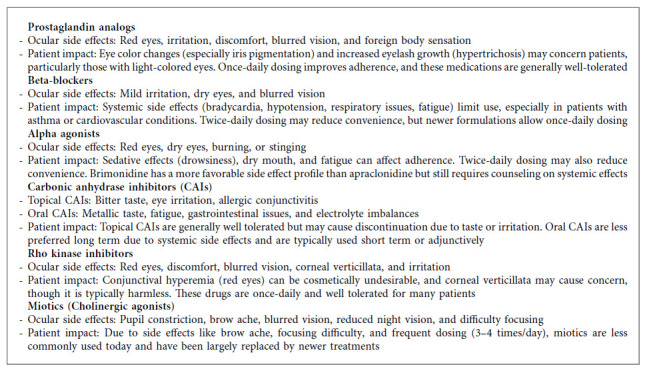
Side effects of glaucoma medications.

**Table 1:** Summary of laser and surgical treatments for glaucoma.

Treatment category	Procedure	Indication/mechanism	Notes/advantages
Laser therapy	Laser trabeculoplasty (ALT, SLT)	OAG; enhances AH outflow	Noninvasive; alternative to surgery; may require repeat treatments
	Laser peripheral iridotomy (LPI)	PACG; creates opening in peripheral iris to relieve pupillary block	First-line for ACG; restores ACA anatomy
	Laser iridoplasty	Adjunct in ACG; contracts peripheral iris to widen angle	Used when LPI alone is insufficient
	Cyclodestructive procedures (transscleral or endoscopic cyclophotocoagulation)	Advanced or refractory glaucoma; partially ablates ciliary body to reduce AH production	Reserved for severe or failed cases; may not provide long-term IOP control

Surgical options	Trabeculectomy	OAG or ACG when other treatments fail; creates new drainage passage	Gold standard; risk of infection, cataract, revision surgery
	Nonpenetrating deep sclerectomy	Alternative to trabeculectomy with fewer complications	Less effective IOP reduction in advanced cases
	Glaucoma drainage implants (Ahmed, Baerveldt)	Refractory glaucoma; provides alternative drainage route	Used after failed trabeculectomy; complications include tube obstruction
	Tube shunt surgery	Similar to drainage implants; long-term management needed	Often secondary procedure
	MIGS: iStent, Hydrus Microstent, Xen Gel Stent, CyPass Micro-Stent, Trabectome	Mild to moderate glaucoma; bypass TM or create new drainage pathways	Less invasive, quicker recovery, safer; balance of efficacy and safety
	Goniotomy	Primarily congenital/juvenile glaucoma; may help open angle in certain ACG cases	Less common in adults
	Cyclocryotherapy	Refractory cases; reduces AH production by freezing ciliary body	Less used today; replaced by more precise cyclophotocoagulation

*Note:* IOP, intraocular pressure.

Abbreviations: ACA, anterior chamber angle; ACG, angle-closure glaucoma; AH, aqueous humor; ALT, argon laser trabeculoplasty; LPI, laser peripheral iridotomy; MIGS, minimally invasive glaucoma surgery; OAG, open-angle glaucoma; PACG, primary angle-closure glaucoma; SLT, selective laser trabeculoplasty; TM, trabecular meshwork.

## Data Availability

Data sharing is not applicable to this article as no datasets were generated or analyzed during the current study.
